# The Exosomal miR-1246 of Laryngeal Squamous Cell Carcinoma Induces Polarization of M2 Type Macrophages and Promotes the Invasiveness of Laryngeal Squamous Cell Carcinoma

**DOI:** 10.1155/2022/4424221

**Published:** 2022-09-26

**Authors:** Lifeng Wu, Na Zuo, Shuo Pan, Yue Wang, Qixue Wang, Jun Ma

**Affiliations:** Department of Otolaryngology-Head and Neck Surgery, Yijishan Hospital of Wannan Medical College, No. 2 Zheshan West Road, Wuhu, 241001 Anhui, China

## Abstract

**Background:**

The possible role and detailed mechanisms of Tumor-associated macrophages (TAMs) in laryngeal squamous cell carcinoma (LSCC) have not been revealed.

**Methods:**

The expressions of typical markers were evaluated by qRT-PCR. In macrophages cocultured with TU212 cells, CD163, and CD206 protein expressions were detected by western blot analysis; IL-10 and IL-12 expressions were detected by ELISA assay. Exosomes isolated from TU212 cells were characterized by TEM analysis. As for the TU212 cells cocultured with macrophages processed with HOK or TU212 cells derived exosomes, their viability, migration, and invasion were assessed by CCK-8 assay, wounding healing, and Transwell assays, respectively.

**Results:**

In this study, macrophages processed with exosomes from human TU212 cells notably advanced LSCC cell viability, migration, and invasion. miR-1246 inhibitor suppressed the M2 polarization of macrophages. Macrophages transfected with miR-1246 inhibitor suppressed LSCC cell viability, migration, and invasion.

**Conclusion:**

In summary, our data implied that the exosomal, miR-1246 of LSCC, induced polarization of M2 type macrophages and promoted the progression of LSCC. This trial is registered with 2020-13.

## 1. Introduction

As a kind of head and neck malignancy, laryngeal cancer has the highest incidence rate in Northeast China and shows an upward trend at a rate of 25% annually, of which more than 95% are laryngeal squamous cell carcinoma (LSCC) [1]. Through continuous exploration and practice, the treatment of LSCC has been standardized as a comprehensive treatment combining surgery with local radiotherapy and/or systemic chemotherapy [2]. Therefore, the effective rate and laryngeal preservation rate of early LSCC treatment have been greatly improved, reaching more than 80%. However, 5-year survival and laryngeal preservation rate of advanced LSCC have not been significantly improved [3]. The occurrence and development of LSCC are the results of the joint action of multiple factors, stages, and genes [4]. At present, the research on the gene level of LSCC is not perfect, and there are few clear therapeutic targets [5]. Thus, the research on the molecular level of LSCC, the early gene diagnosis, and the development of new treatment methods all need us to have a further understanding of the pathogenesis of LSCC, to realize the gene-level treatment of LSCC patients, make up for the shortcomings of traditional surgery and radiotherapy and chemotherapy, and improve the quality of LSCC patients' life.

Extracellular vesicles (EVs) can be released by cancer cells and all other cells into the extracellular space and communicate with adjacent or distal cells. EVs are surrounded by lipid bilayers containing protein and nucleic acid substances, released from cells in the physiological and pathological environment and reach a closer or farther distance by entering the circulatory system [6]. Exosomes are derived from the multivesicular bodies (MVBs)' membrane invagination with a diameter of 30-150 nm [7]. Exosomes are secreted outside cells after fusing MVBs with the plasma membrane and are rich in a series of molecules, including protein, lipid, DNA, and RNA [8]. Besides, exosomes contain various RNAs, such as snRNA, miRNA, mRNA, tRNA, rRNA, piRNA, lncRNA, and small nucleolar RNA [9]. Because exosomes carry surface molecules, which can provoke signal transduction via receptor-ligand interaction or can internalize through phagocytosis and endocytosis, and even transfer the contents into them through membrane fusion with receptor cells [10]. Therefore, donor cells' exosomes can change the state of physiological and pathological in recipient cells.

The tumor microenvironment (TME) is composed of an extracellular matrix (ECM), immune cells, and stromal cells [11]. TME has been found to determine abnormal tissue function and exert a crucial role in subsequent malignant tumor evolution. With the related remodeling of TME, tumor cells continue to proliferate and increase in size [12]. As one of the most abundant stromal cell types in the tumor environment, macrophages can be triggered to exhibit the M1 phenotype promoting tumor immunity or the M2 phenotype promoting tumor inflammation [13]. Similar to M2 macrophages, tumor-associated macrophages (TAMs) advance tumor metastasis through the enhancement of tumor cell movement, promotion of angiogenesis, and degradation of the extracellular basement layer [14]. TAMs have been considered an essential factor in tumor progression [15]. At present, the drivers behind TAMs differentiation remain unknown, and it is also unclear if tumor-derived exosomes are necessary for TME changes. Therefore, this study was designed to identify the microenvironmental mechanisms to form TAMs via exosome-mediated communication between immune cells and cancer cells, which will provide ideas for predictive markers and targeted treatment for advanced LSCC.

## 2. Materials and Methods

### 2.1. Clinical Sample

Fifteen LSCC tissues and plasma were obtained from Yijisan Hospital of Wannan Medical College. All experimental protocols were permitted by the ethics committee. Each participant signed the written informed consent.

### 2.2. Cell Culture and Transfection

Human LSCC cells TU212, oral keratinocytes (HOK) together with THP-1 cells were collected from American Type Culture Collection (ATCC, Manassas, VA, USA), cultivated within DMEM (Thermo Fisher Scientific, Waltham, MA, USA) that contained 10% fetal bovine serum (FBS, Gibico, NY, USA) and incubated in humid under 5% CO_2_ and 37°C conditions.

Genechem (Shanghai, China) was responsible for constructing the NC inhibitor and miR-1246 inhibitor. Each of the above plasmids was individually transfected in cells via Lipotransfectamine 3000 (Thermo Fisher Scientific, Waltham, MA, USA) in line with the instruction.

### 2.3. Macrophage Differentiation

After being treated with 185 ng/mL phorbol ester (PMA, dissolved with DMSO) for 6 h, THP-1 cells were induced to differentiate into macrophages (M0). Then, macrophages were cultivated with 20 ng/mL IFN-*γ* and 100 ng/mL LPS, respectively, for more than 48 h to polarize macrophages to the M1 phenotype. In addition, macrophages were fostered with IL-4 and IL-13 (20 ng/mL for each) for more than 48 h to polarize them to the M2 phenotype [16].

### 2.4. Extraction of Exosomes

Cell culture supernatant collected from different groups or plasma was treated with 10 min centrifugal at 300 × *g*, and then assimilated, followed by centrifugal at 2, 000 × *g* (10 min) and 10, 000 × g (30 min) to dislodge shedding vesicles. Further, removed the supernatant by 90 min ultracentrifugation at 140, 000 × *g*, and obtained the precipitate exosomes. The precipitate was washed with PBS buffer, resuspended, centrifuged for 90 min at 140, 000 × *g*, resuspended with 100 *μ*L PBS buffer, and frozen at -80°C for standby.

### 2.5. Transmission Electron Microscopy (TEM) Assay

The exosomes at 0.5 mg/mL concentration were obtained by ultracentrifugation and added to PBS buffer solution. The exosome suspension was dropped on the copper plate, placed on the filter paper, and then lighted with an incandescent lamp for 10 min. The exosomes were incubated with 1% phosphotungstic acid for 5 min, lighted for another 20 min, and observed via transmission electron microscope (FEI TECNAI G20, USA).

### 2.6. Fluorescent Labeling and Transfer of Exosomes

The extracted exosomes from TU212 were incubated with PKH26 (Sigma-Aldrich, MO, USA), cocultivated for 48 h with macrophages cells, and then stained with DAPI. A laser confocal microscope (Leica SP2, Germany) was used to determine whether macrophages could endocytose the exosomes from LSCC cells.

### 2.7. Coculture

TU212 cells were inoculated onto Transwell culture inserts with 0.4 *μ*m pore size (Corning, USA) and then transferred to dishes inoculated with macrophages.

### 2.8. Enzyme-Linked Immunosorbent Assay (ELISA)

Based on specific protocols, ELISA kits (San Diego, CA) were carried out to analyze the cytokine concentrations, such as human IL-10 and IL-12, isolated in macrophages [17].

### 2.9. CCK-8 Assay

TU212 cells (5 × 10^3^/well) were inoculated and cocultured with macrophages treated with exosomes from HOK and TU212 cells or transfected macrophages with a 24, 48, or 72 h CCK-8 kit (Sigma, USA). A spectrophotometer (Molecular Devices, San Jose, USA) was utilized to measure the absorbance (OD) at 450 nm.

### 2.10. Wound Healing Assay

Cultured TU212 cells at the appropriate density, and scratched a wound by 200 *μ*L pipette tip when the cells had reached 80% confluence. Then, TU212 cells were cocultured with macrophages treated with exosomes from HOK and TU212 cells or transfected macrophages. Finally, observed cell images at 0 and 48 h (200×) with an inverted microscope (Olympus, Japan).

### 2.11. Transwell Analysis

Trypsinized TU212 cells into a single cell suspension and washed 3 times, and then cocultured with macrophages treated with exosomes from HOK and TU212 cells or transfected macrophages. For Transwell invasion assays, prediluted Matrigel with serum-free DMEM (1: 3), and used polycarbonate film to uniformly covered it in the Transwell chamber at 37°C for 1 h. The lower Transwell chambers were handled with 48 h incubation at 37°C with a medium including 10% FBS. The migratory and invasive cells were dyed with 0.1% crystal violet and photoed by a microscope (Olympus, Japan) (200×).

### 2.12. qRT-PCR Assay

Extracted total RNA from cells or exosomes, and prepared cDNA through reverse transcription with the RNeasy plus micro kit, followed by a qRT-PCR experiment using Step One System (Life Technologies Corp). By Primer Premier software 4.0 (Premier, Canada), all primer sequences were designed and shown in Table 1. *β*-actin was normalized by 2^−ΔΔCT^approach [18].

### 2.13. Western Blotting Assay

Protein was separated from macrophages, determined by a BCA kit (Beyotime Biotechnology, China), extracted with 12% SDS-PAGE, and transferred into PVDF membranes (Millipore, USA). Next, membranes were cultured in 5% skimmed milk, incubated with primary antibodies overnight under 4°C, rinsed, followed by 1 h incubation under ambient temperature with HRP-labeled secondary antibody (1: 4,000, SA00004-10, Proteictech, China). Finally, observed protein blots with the enhanced chemiluminescence kit (ECL, Millipore, Bedford, USA) and quantified by ImageJ software (NIH, version 4.3). All used primary antibodies included anti-CD206 (1: 2, 000, 18704-1-AP, Proteictech, China), anti-CD163 (1: 2, 000, 16646-1-AP, Proteictech, China), and anti-*β*-actin (1: 5, 000, 66009-1-Ig, Proteictech, China), with *β*-actin being the endogenous control.

### 2.14. Statistical Analysis

Conducted data analysis through GraphPad Prism 5.0 and expressed data as mean ± SD. Differences between the two groups were analyzed using *t*-test. One way and two-way ANOVA and Tukey's poc host analysis were used to compare the differences between groups. *P* < 0.05 represented statistical significance.

## 3. Results

### 3.1. TU212 Cells Induce M2 Polarization of Macrophages

**Figure 1:**
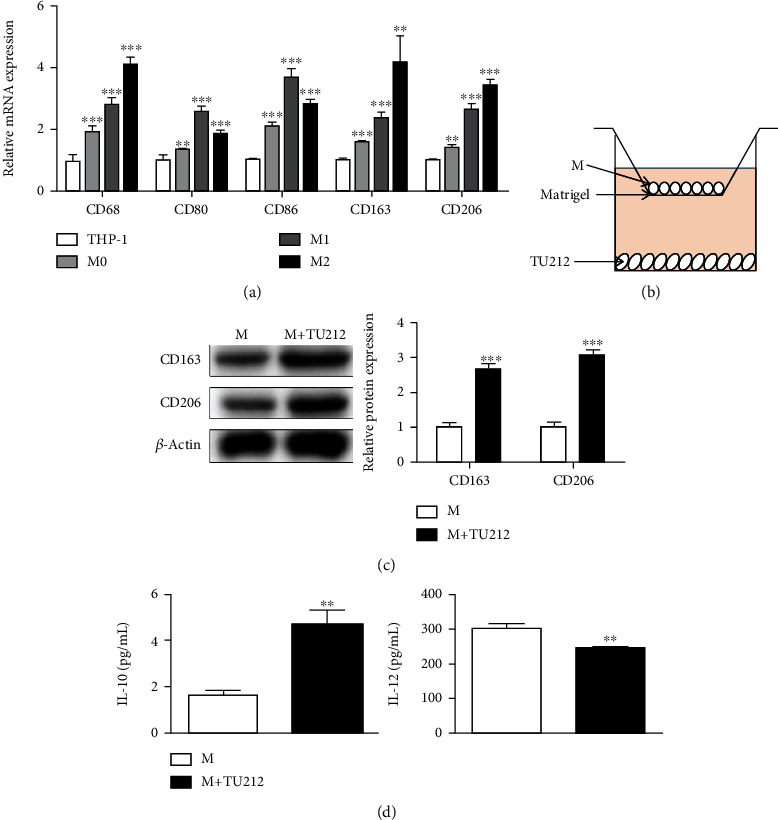
TU212 cells trigger the M2 polarization of macrophages. (a) The expressions of typical markers of M0 (CD68), M1 (CD86 and CD80), and M2 (CD163 and CD206) were evaluated by qRT-PCR. THP-1 cells. (b) Coculture of macrophages and TU212 cells. In macrophages cocultured with TU212 cells, the protein expressions of CD163 and CD206 were detected by western blot analysis (c); IL-10 and IL-12 levels were detected by ELISA assay (d). ^∗∗^*P* < 0.01, ^∗∗∗^*P* < 0.001*vs*. THP-1 or M. M: Macrophages.

THP-1 cells were induced to differentiate into M0, M1, and M2 macrophages, respectively, as described previously [16]. Then western blot was implemented to measure macrophage markers' expression levels of M0 (CD68), M1 (CD80 and CD86), and M2 (CD163 and CD206), as shown in Figure 1(a). Then macrophages were cocultured with TU212 cells displayed in Figure 1(b). The protein expressions of M2 macrophage markers (CD163 and CD206) were evaluated by western blot. Based on Figure 1(c), CD163 and CD206 protein levels were obviously upregulated in the coculture of macrophages and TU212 cells compared to that in macrophages alone. In addition, ELISA analysis was used to assess IL-10 and IL-12 expressions.  Figure 1(d) indicated that IL-10 was increased, while IL-12 was decreased in coculture of macrophages and TU212 cells relative to that in macrophages alone.

### 3.2. Exosomes Derived from TU212 Cells Induce M2 Polarization of Macrophages

**Figure 2:**
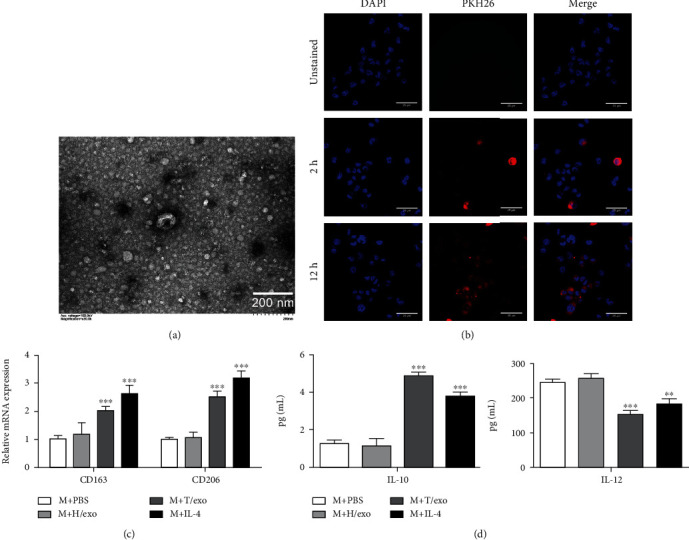
Exosomes derived from TU212 cells provoke the M2 polarization of macrophages. (a) Exosomes isolated from TU212 cells were characterized by TEM analysis (scale bar: 200 nm). (b) The internalization of exosomes derived from TU212 cells by macrophages was evaluated by PKH-26 staining (scale bar: 25 *μ*m). (c) The protein expressions of CD163 and CD206 in macrophages treated with HOK cell- or TU212 cell-derived exosomes were tested by western blot. (d) The expressions of IL-10 and IL-12 in macrophages treated with HOK cell- or TU212 cell-derived exosomes were determined by ELISA assay. ^∗∗^*P* < 0.01, ^∗∗∗^*P* < 0.001*vs*. M + PBS. M: Macrophages.

To testify whether TU212 cell-derived exosomes could be absorbed into macrophages, we first collected exosomes from a TU212 cell conditioned medium. In Figure 2(a), TEM disclosed a cup-shaped morphology for the purified exosomes. To estimate the biological relationship between macrophages and exosomes derived from TU212 cells, macrophages were cultured with fluorescently labeled exosomes for 2 h and 12 h, and stained exosomes were found to exist in the cytoplasm of macrophages by confocal microscopy (Figure 2(b)). To confirm the promotion effect of TU212 cell-derived exosomes on M2 macrophage polarization, the typical marker expressions of the M2 phenotype were detected by qRT-PCR analysis. According to Figure 2(c), compared with the PBS treatment group, there was no obvious difference in the mRNA expressions of CD163 and CD206 within the HOK-exosomes treatment group. While macrophages were treated with exosomes derived from TU212 cells, the CD163 and CD206 expressions were obviously upregulated (Figure 2(c)). Similarly, ELISA data illustrated that after treatment with exosomes derived from HOK cells, there was no significant difference in IL-10 and IL-12 expressions, while exosomes derived from TU212 cells notably upregulated the IL-10 expression, while downregulated the IL-12 expression displayed in Figure 2(d).

### 3.3. Macrophages Induced with Exosomes Derived from TU212 Cells Facilitate the Viability, Migration, and Invasion of TU212 Cells

**Figure 3:**
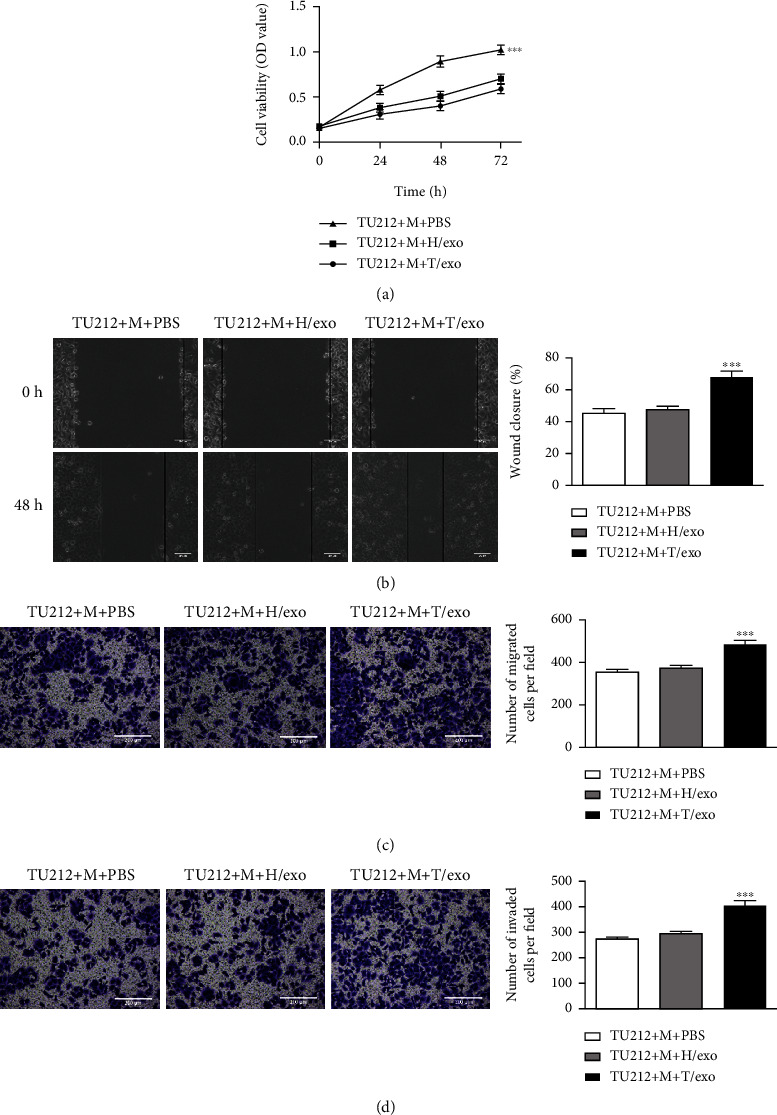
Macrophages induced with exosomes derived from TU212 cells promote TU212 cell viability, migration, and invasion. The viability, migration, and invasion of TU212 cells cocultured with macrophages treated with exosomes derived from HOK or TU212 cells were assessed by CCK-8 assay (a) wounding healing (scale bar: 200 *μ*m) (b) Transwell migration (scale bar: 200 *μ*m), and (c) invasion (scale bar: 200 *μ*m) (d) assays ^∗∗∗^*P* < 0.001*vs*. TU212 + M + PBS. M: Macrophages.

To further investigate the functions of macrophages treated with TU212 cell-derived exosomes on TU212 cells, firstly CCK-8 assay was performed. According to Figure 3(a), TU212 cell viability had no notable difference between the PBS treatment group and HOK-exosomes treatment group, while obviously promoted over time after cocultured with macrophages treated with exosomes derived from TU212 cells (Figure 3(a)). In addition, wound healing, and Transwell analysis were adopted to evaluate TU212 cell migration and invasion. Based on Figures 3(b)to 3(d), relative to the PBS treatment group, the number of migrated and invasive TU212 cells showed obvious difference after cocultured with macrophages incubated with HOK-exosomes, while was notably increased after TU212 cells cocultured with macrophages treated with TU212 cell-derived exosomes.

### 3.4. TU212 Cell-Derived Exosomes Convey miR-1246 to Macrophages

**Figure 4:**
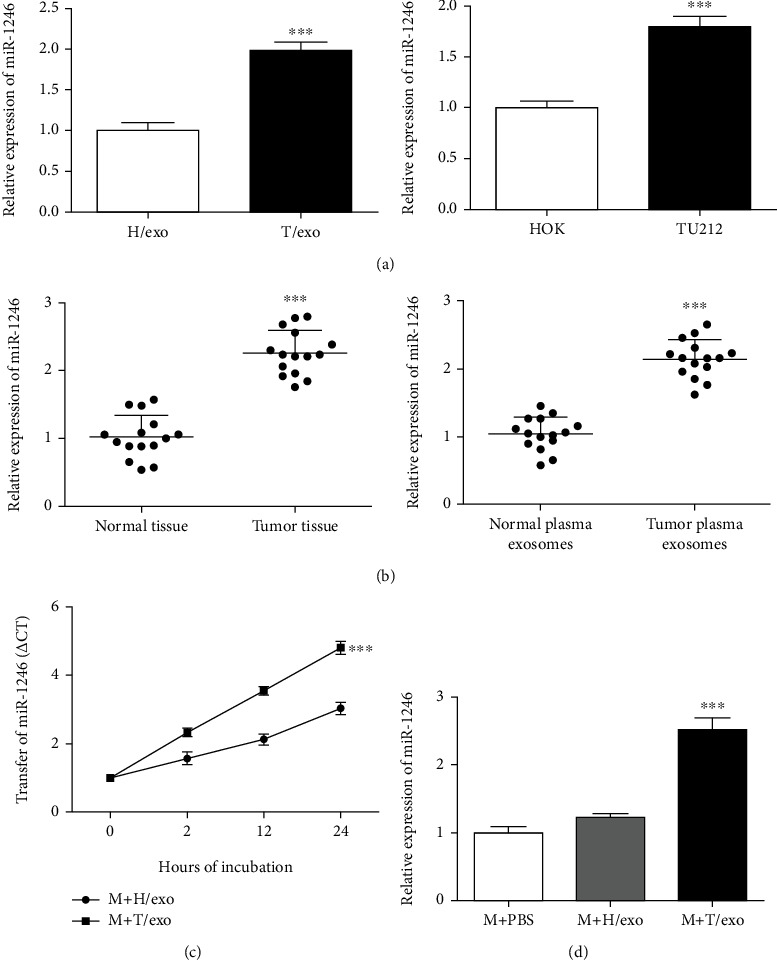
TU212 cell-derived exosomes deliver miR-1246 to macrophages. (a) The miR-1246 expressions in HOK and TU212 cells or their corresponding exosomes were accessed by qRT-PCR assay. ^∗∗∗^*P* < 0.001*vs*. HOK cells or H/exo. (b) The miR-1246 expressions in LSCC tissues or the corresponding exosomes were determined by qRT-PCR assay. ^∗∗∗^*P* < 0.001*vs*. Normal tissues or Normal plasma-derived exosomes. (c) The miR-1246 expression in macrophages treated with HOK cell- or TU212 cell-derived exosomes at indicated times was assessed by qRT-PCR assay. ^∗∗∗^*P* < 0.001*vs*. M + H/exo. (d) The miR-1246 expression in macrophages treated with HOK cell- or TU212 cell-derived exosomes was assessed by qRT-PCR assay. ^∗∗∗^*P* < 0.001*vs*. M + PBS. H/exo: HOK cell-derived exosomes; T/exo: TU212 cell-derived exosomes; M: Macrophages.

Bioactive molecules contained in exosomes participate in intercellular communication [19]. miR-1246 has been reported highly expressed in LSCC. To clarify miR-1246 expression in TU212 cell-derived exosomes derived and LSCC plasma, qRT-PCR analysis was conducted. Based on Figure 4(a), higher miR-1246 expression presented in TU212 cells and TU212 cell-derived exosomes compared to that in HOK cells and HOK cell-derived exosomes, respectively. In addition, according to Figure 4(b), miR-1246 expression was upregulated within LSCC tissues and LSCC plasma exosomes relative to that in normal tissues and normal plasma exosomes. Furtherly, Figures 4(c) and 4(d) showed higher miR-1246 expression in macrophages processed with TU212 cell-derived exosomes, suggesting that tumor-derived exosomes could convey miR-1246 to macrophages.

### 3.5. Macrophages Transfected with miR-1246 Inhibitor Suppress TU212 Cell Viability, Migration, and Invasion

**Figure 5:**
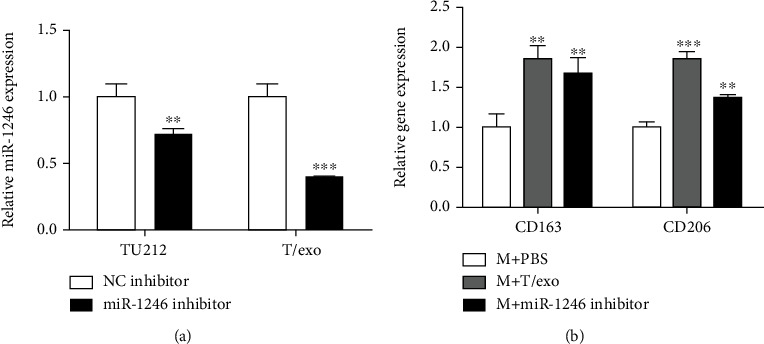
miR-1246 effects on M2 polarization of macrophages. (a) The miR-1246 expressions in miR-1246 inhibitor transfected TU212 cells and the corresponding exosomes were detected by the qRT-PCR assay. ^∗∗^*P* < 0.01, ^∗∗∗^*P* < 0.001*vs*. NC inhibitor. (b) The CD163 and CD206 expressions in miR-1246 inhibitor-transfected macrophages were detected by western blot. ^∗∗^*P* < 0.01, ^∗∗∗^*P* < 0.001*vs*. M + PBS. M: Macrophages.

**Figure 6:**
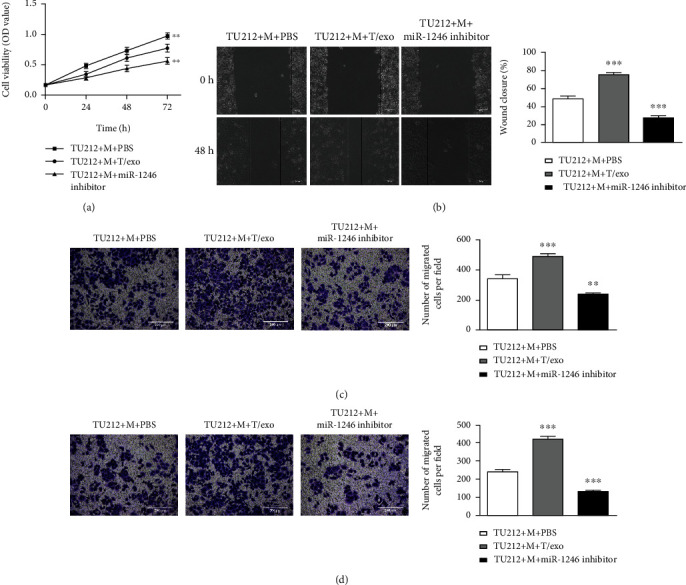
Macrophages transfected miR-1246 inhibitor inhibits the viability, TU212 cell migration, and invasion. (a) The viability, migration, and invasion of TU212 cells cocultured with miR-1246 inhibitor-transfected macrophages were detected by CCK-8 assay, wounding healing (scale bar: 200 *μ*m) (b) Transwell migration (scale bar: 200 *μ*m), and (c) invasion (scale bar: 200 *μ*m) (d) assays ^∗∗^*P* < 0.01, ^∗∗∗^*P* < 0.001*vs*. TU212 + M + PBS. M: Macrophages.

Furthermore, the miR-1246 effects in macrophages on TU212 cell functions were further investigated. Firstly, the miR-1246 inhibitor was transfected into TU212 cells, and the miR-1246 expression was assessed by qRT-PCR assay. According to Figure 5(a), miR-1246 expression was inhibited in miR-1246 inhibitor-transfected TU212 cells and corresponding exosomes. Moreover, the miR-1246 inhibitor notably suppressed the M2 polarization of macrophages by decreasing CD163 and CD206 expressions (Figure 5(b)). Moreover, the influences of miR-1246 inhibitor-transfected macrophages on TU212 cell viability, migration, and invasion were further investigated by CCK-8, wound healing, and Transwell assays. Based on Figures 6(a)–6(d), macrophages transfected miR-1246 inhibitor remarkably restrained TU212 cell viability, migration, and invasion.

## 4. Discussion

TME is a complex ecosystem and an active participant in all stages of LSCC occurrence and development [20]. TME is composed of many cell types, which can regulate too many cell-cell interactions, further playing a significant impact on cancer development, progression, and treatment [21]. As one cell type with abundant stromal in TME, macrophages are highly plastic and can be activated into M1 or M2 polarization by stimulation of different chemokines and cytokines from TME [22]. Studies have shown that M1 macrophages can enhance innate and adaptive immunity. M2 macrophages, also known as TAMs, have immunosuppressive effects and widely exist in advanced cancer, helping to enhance tumor metastasis and invasion [23]. Moreover, M1 and M2 phenotypes can be converted to each other [24]. In addition, M2 macrophages can be further classified as M2a, M2b, and M2c, which mainly depend on the stimulation of different factors, and the activation of M2c is a response to IL-10 and IL-12 [25, 26]. M2c is usually used as inactivated macrophages because their common markers are the downregulation of proinflammatory cytokines, the elimination of abnormal activity, and the continuation of delayed functional programs [27]. In TME, TAMs characterized by the poor ability of antigen presentation, can block T cell proliferation, and inhibit NK cell activation by regulating IL-10 and IL-12, which is helpful to the inhibition of the immunosuppressive environment [28–30]. In our study, after coculture with TU212 cells, the protein expressions of CD163 and CD206 were notably increased, the expression of IL-10 was promoted, and the expression of IL-12 was downregulated. These data suggested that TU212 cells notably induced macrophage M2 polarization.

In TME, the movement of cancer cells is often affected by exosomes [31]. Interestingly, different integrin expression patterns were shown in exosomes from different tumor types, which can influence organ-specific metastasis. The premetastasis niche can be prepared through organ-specific cells ingesting cancer cell-derived exosomes. Studies have shown that exosome integrin *α*6*β*1 and *α*6*β*4 are involved in the process of lung metastasis. In contrast, liver metastasis is related to the expression of exosome integrin *α*v*β*5, suggesting that exosome integrin can be used to predict organ-specific metastasis [32]. In addition to the formation of a premetastatic niche, the exosomes released by cancer cells also directly result in early metastasis [33]. Therefore, tumor cell-derived exosomes participate in the potential complex signal transduction network between TME stromal cells and distal organs [34]. Moreover, clinical studies have shown that TAMs supply important survival factors and protumorigenesis, ECM modifying enzymes, and proangiogenic factors [35]. Exosomes from cancer cells can enhance the persistence and induction of inflammation, which is functionally conducive to the progress of the disease [36]. In the present study, TU212 cell-derived exosomes notably induced M2 polarization of macrophages. Functionally, after macrophages were treated with TU212 cell-derived exosomes, TU212 cell viability, migration, and invasion were obviously promoted.

Overexpressed miR-1246 has been found in various human cancer types, including colorectal cancer (CRC), gastric cancer, prostate cancer, and so on [37–39]. Moreover, miR-1246 is overexpressed in LSCC tissues [40]. It has been demonstrated recently that exosomes selectively loaded or retained specific miRNAs, suggesting obvious exosomal enrichment of specific miRNAs relative to most cellular miRNAs [41]. For example, exosomal miR-21 secreted from bladder cancer cells promotes cancer progression via activating the PI3K/AKT pathway in macrophages [42]. Exosomal miR-934 from tumor cells promotes colorectal cancer liver metastases via initiating macrophage M2 polarization [43]. This trial found enriched miR-1246 in exosomes derived from LSCC tissues and TU212 cells. Furthermore, exosomes transferred miR-1246 to macrophages (M2 macrophage polarization) from TU212 cells and subsequently accelerated tumor migration and invasion, indicating that a more aggressive phenotype in these cells can be conferred by elevated miR-1246 level in macrophages. Moreover, the miR-1246 inhibitor was successfully transfected into macrophages. Macrophages transfected miR-1246 inhibitor remarkably inhibited the viability and metastasis of TU212 cells. Thus, exosomal miR-1246 seemed to be involved in TME formation and play a vital role in subsequent LSCC cell migration and invasion.

Despite a lot of work, the current research still has limitations. Only TU212 cells were used in this study. Different types of LSCC cell lines can reflect differences of LSCC cell-derived exosomes on macrophage polarization, further affecting LSCC progression, which needs to be further investigated. To sum up, exosomal miR-1246 of LSCC induced polarization of M2-type macrophages and promoted the progression of LSCC.

## 5. Conclusion

In this study, macrophages treated with exosomes from a human TU212 cell conditioned medium notably promoted LSCC cell viability, migration, and invasion. miR-1246 inhibitor inhibited the M2 polarization of macrophages. miR-1246 inhibitor-transfected macrophages suppressed LSCC cell viability, migration, and invasion. These data suggested that the exosomal miR-1246 of LSCC induced polarization of M2 type macrophages and promoted the progression of LSCC.

## Figures and Tables

**Table 1:** Primer sequences.

Gene name	Primer sequences
CD68	F: 5′-CATTCCCCTATGGACACCTCA-3′
	R: 5′-GTCTCCGGATGATGCAGAAAG-3′
CD80	F: 5′-CAACCACAGCTTCATGTGTCTCA-3′
	R: 5′-TGAGATTAAGGTAATGGCCCAGGA-3′
CD86	F: 5′-AGGGAGGGGTTTTGGTG-3′
	R: 5′-CCGTAGGACATCTGTAGGCT-3′
CD163	F: 5′-TTTGTCAACYYGAGTCCCTTCAC-3′
	R: 5′-TCCCGCTACACTCGTTTTCAC-3′
CD206	F: 5′-CATATCGGGTTGAGCCACTT-3′
	R: 5′-GAGGGATCTCCTGTGTTCCA-3′
*β*-actin	F: 5′-CCTGGCACCAGCACAAT-3′
	R: 5′-GGGCCGGACTCGTCATAC-3′

## Data Availability

The raw data supporting the conclusions of this manuscript will be made available by the authors, without undue reservation, to any qualified researcher.
